# “Gestalt diagnosis” for children with suspected genetic syndromes

**DOI:** 10.1186/1824-7288-41-S2-A16

**Published:** 2015-09-30

**Authors:** Paola Cianci, Angelo Selicorni

**Affiliations:** 1 Pediatric Genetic Unit, Pediatric Department, Milano Bicocca University, Monza, 20900, Italy; 2 Pediatric Department, Insubria University, Varese, 21100, Italy

## 

Gestalt identification is the process by which healthcare practitioners actively organize clinical perceptions into specific diagnostic ideas. This implies that clinicians, in particular geneticists, have the ability to quickly generate diagnostic hypotheses in absence of complete information, simply on the basis of combination of specific facial dysmorphisms or particular elements of the clinical history. This is particularly frequent in clinical genetics because the “facial dysmorphisms” of the patient represent often the most specific clinical criteria of the syndrome itself. Down [1], Cornelia de Lange [2], Wolf-Hirshhorn [3], Noonan [4], Rubistein-Taybi [5] Kabuki [6], Treacher Collins [7] and Williams syndrome [8] are examples well known to pediatricians but this is true for the great majority of genetic syndromes. In these situations facial dysmrphisms can have variable expression between different patients (also in the same family) or can become more or less evident over time. Moreover the “gestalt process” can also be applied in case of specific auxologic and/or neurologic evolution. In patients with Prader-Willi syndrome (PWS) in the first months of life hypotonia is evident and tends to improve between 8 and 11 months of age; poor growth and feeding problems are frequent too. Later on hypotonia improves and feeding difficulties are replaced by hyperphagia and obesity. This typical evolution can permit to suspect in every phase PWS diagnosis and to perform methylation test for 15q11.2 region [9]. Some neuro pediatricians suggest that Angelman syndrome's EEG pattern is typical enough to be recognized by sensitized professionals [10]. A specific behavioral phenotype, including significant sleep disturbance, stereotypies and oppositional behavior, is pretty characteristic of Smith-Magenis syndrome [11]. Again, PTEN gene testing may be considered for patients with extreme macrocephaly associated with intellectual disability/autistic behaviour [12]. Within overgrowth syndrome, Beckwith-Wiedemann syndrome (BWS) can be easily recognized thanks to the association between hemi-hypertrophy, macroglossia, abdominal wall defects and frontal nevus flammeus [13]. Finally within patients with short stature the evidence of disproportion between crown-rump and overall lengths, or between limbs and trunk univocally direct the diagnosis toward the skeletal dysplasias [14].

In conclusion the knowledge of facial features and/or natural history (auxological, neurological behavioural etc) of the most common genetic syndromes can help pediatricians in hypothesizing them through a “gestalt process” in order to quickly confirm the clinical diagnosis with the specific genetic tests.
